# Innate Lymphoid Cells Promote Recovery of Ventricular Function After Myocardial Infarction

**DOI:** 10.1016/j.jacc.2021.07.018

**Published:** 2021-09-14

**Authors:** Xian Yu, Stephen A. Newland, Tian X. Zhao, Yuning Lu, Andrew S. Sage, Yanyi Sun, Rouchelle S. Sriranjan, Marcella K.L. Ma, Brian Y.H. Lam, Meritxell Nus, James E. Harrison, Simon J. Bond, Xiang Cheng, Jean-Sébastien Silvestre, James H.F. Rudd, Joseph Cheriyan, Ziad Mallat

**Affiliations:** a Department of Medicine, Division of Cardiovascular Medicine, University of Cambridge, Cambridge, United Kingdom; b Department of Cardiology, Union Hospital, Tongji, Medical College, Huazhong University of Science and Technology, Wuhan, China; c Université de Paris, PARCC, INSERM, F-75015 Paris, France; d The Wellcome Trust-MRC Institute of Metabolic Science-Metabolic Research Laboratories, University of Cambridge, Cambridge, United Kingdom; e Division of Experimental Medicine and Immunotherapeutics, University of Cambridge, Cambridge, United Kingdom; f Cambridge Clinical Trials Unit, Cambridge University Hospitals NHS Foundation Trust, Cambridge, United Kingdom

**Keywords:** cytokines, heart failure, innate lymphoid cells, interleukin-2, lymphocytes, myocardial infarction, ACS, acute coronary syndrome, Arg1, Arginase 1, IL, interleukin, ILC2, type 2 innate lymphoid cells, LAD, left anterior descending, MI, myocardial infarction, PcAT, peri-cardial adipose tissue, RNAseq, RNA sequencing, Treg, regulatory T cells

## Abstract

**Background:**

Innate lymphoid cells type 2 (ILC2s) play critical homeostatic functions in peripheral tissues. ILC2s reside in perivascular niches and limit atherosclerosis development.

**Objectives:**

ILC2s also reside in the pericardium but their role in postischemic injury is unknown.

**Methods:**

We examined the role of ILC2 in a mouse model of myocardial infarction (MI), and compared mice with or without genetic deletion of ILC2. We determined infarct size using histology and heart function using echocardiography. We assessed cardiac ILC2 using flow cytometry and RNA sequencing. Based on these data, we devised a therapeutic strategy to activate ILC2 in mice with acute MI, using exogenous interleukin (IL)-2. We also assessed the ability of low-dose IL-2 to activate ILC2 in a double-blind randomized clinical trial of patients with acute coronary syndromes (ACS).

**Results:**

We found that ILC2 levels were increased in pericardial adipose tissue after experimental MI, and genetic ablation of ILC2 impeded the recovery of heart function. RNA sequencing revealed distinct transcript signatures in ILC2, and pointed to IL-2 axis as a major upstream regulator. Treatment of T-cell–deficient mice with IL-2 (to activate ILC2) significantly improved the recovery of heart function post-MI. Administration of low-dose IL-2 to patients with ACS led to activation of circulating ILC2, with significant increase in circulating IL-5, a prototypic ILC2-derived cytokine.

**Conclusions:**

ILC2s promote cardiac healing and improve the recovery of heart function after MI in mice. Activation of ILC2 using low-dose IL-2 could be a novel therapeutic strategy to promote a reparative response after MI.

The mechanisms that lead to heart failure post-myocardial infarction (MI) are initiated very early after ischemic injury, triggering waves of immune responses that play critical roles in tissue healing and remodeling (1). None of the current therapies aimed at improving cardiac remodeling directly target the immune system around the time of ischemic injury (2). Therefore, important advances in therapeutic management could be expected from a better understanding and early targeting of the post-ischemic immune response.

During the initial inflammatory phase after ischemic injury, the rapid infiltration of neutrophils to the infarct area begins the process of clearing the necrotic tissue. This is aided by Ly6C^hi^ monocytes recruited through secretion of chemokines such as CCL2 and CCL7, which in turn potentially differentiate into activated macrophages. The macrophages are further conditioned during the subsequent regulatory phase (3), shifting the balance from proinflammatory classical activation toward the alternative activation phenotype, which is more conducive for tissue repair (4, 5, 6, 7). Circulating CD4^+^ T cells infiltrate the wounded heart early on and their presence helps to slew the macrophage phenotype toward the alternative activation phenotype (8). During the resolution phase, the activation of regulatory T cells (Tregs) is beneficial, reducing effector T-cell activation, and promoting alternative macrophage activation and tissue repair (9,10).

Type 2 innate lymphoid cells (ILC2s) form a cluster of cells that secrete large amounts of type 2 cytokines, such as interleukin (IL)-5 and IL-13. Morphologically, they are very similar to T cells but lack the ability to express recombined surface antigen receptors, and thus function in an antigen-independent manner. Activation of these cells is primarily through the cytokines IL-25 and IL-33, further supported by lymphocyte-derived IL-2 (11). Although ILC2s are present in secondary lymphoid tissues, they are 10-fold more prevalent in barrier tissues (12,13). In our previous work, we found that the adventitia of arteries and fat-associated lymphoid clusters in perivascular adipose tissue contain ILC2s, which expand in response to atherosclerosis (14). Further work (15) showed that adventitial stromal cells provide a supportive niche in these tissues, supplying them with IL-33 and thymic stromal lymphopoietin. Perivascular ILC2s are critical regulators of macrophage behavior, promoting alternative macrophage activation, thereby limiting the development of unstable atherosclerotic plaques, directly through the secretion of IL-13 (14). ILC2s also accumulate in pericardial fat-associated lymphoid clusters and mediastinum, and a population of IL-33-dependent ILC2s present in human and mouse hearts (16) has been shown to expand in settings of myocarditis, pericarditis, and MI. However, the role of endogenous ILC2s in the regulation of the immune and healing response post-MI remains unexplored.

## Methods

### Experimental mice

All work was conducted under UK Home Office project license regulations after approval by the Ethical Review Committee of the University of Cambridge. The following mouse strains were used: C57BL/6 (in house), *Rag2*^*−/−*^*(*Jax), *Rora*^*fl/fl*^ *Cd127*^*cre*^ *Ldlr*^*−/−*^ (ILC2^KO^), and *Rora*^*fl/fl*^ *Cd127*^*WT*^ *Ldlr*^*−/−*^ (ILC2^WT^) (14,17,18). All experimental mice were females.

### Left anterior descending coronary artery ligation model

Heart failure post-MI is mostly due to abnormal cardiac healing of large infarcts. Therefore, we used the permanent left anterior descending (LAD) ligation model (19). Expansion of ILC2s after MI was achieved using IL-2/jes6-1 (14,20) injected intraperitoneally at 1 μg per mouse 3 times a week for 4 weeks. Soluble ST2 was a kind gift from Suzanne Cohen. Mice received 10 mg/kg soluble ST2 (sST2) intravenously on day 0 (D0) and, D1 to D3 post-MI.

### Echocardiography

Transthoracic echocardiography was performed using Vevo 3100 with an MX400 linear array transducer (VisualSonics) at 30 MHz. Cardiac function was measured on M-mode images.

### Histological analysis

Heart cryostat sections were stained with Masson trichrome to determine scar size. Scar size (in %) was calculated as total infarct circumference divided by total left ventricular circumference. Collagen deposition was determined using Sirius red staining under polarized light (14) and quantified using Fiji (21) as % total area.

### Flow cytometry

Single-cell suspension for spleen, mesenteric lymph nodes, and pericardial and peri-gonadal white adipose tissue was generated (14). Cells were washed in phosphate-buffered saline (PBS) 1% fetal calf serum before labeling with desired antibody cocktails (Supplemental Table 1). Intracellular antigens were detected using the IC Fix/perm kit (BD Biosciences) as per the manufacturer’s instructions. Cells were analyzed on an LSR-Fortessa (BD Biosciences). Subsequent data were analyzed with FloJo X analysis software (FreeStar). Examples of each population gating hierarchy are provided in Supplemental Figure 1.

### Low-input RNA sequencing

A total of 100 ILC2s were sorted directly into single-cell lysis buffer (Takara Bio) and stored at −70ºC until library preparation. The lysate was subjected to direct oligo-dT-based reverse transcription and complementary DNA (cDNA) amplification using SMART-Seq v4 Ultra Low Input RNA Kit (Takara Bio); 150 pg of amplified cDNA was then used to generate sequencing library using Illumina Nextera XT DNA Library Preparation Kit, validated by an Agilent 2100 Bioanalyzer and pooled at equal molar concentrations. Sequencing was performed on an Illumina HiSeq4000 instrument (Single-end 50). An average of approximately 15 million reads were obtained per sample.

### Sequencing bioinformatics

Raw sequence reads were aligned to the mouse GRCm38 genome and gene level counts generated using STAR (v2.5.0a). Differential expression was performed using DeSeq2. Genes were considered differentially expressed when logFC ≥1 and false discovery rate was <0.05. Canonical pathways enrichment predicted upstream regulatory components and network analysis was performed using QIAGEN Ingenuity pathway analysis software (QIAGEN IPA). RNA sequencing (RNAseq) data will be available at the Gene Expression Omnibus.

### Serum cytokine quantification

Mouse IL-5 and IL-13 were detected by enhanced sensitivity CBA flex set (R and D systems), following the manufacturer’s instructions. Human cytokine analysis was performed using a MesoScale Diagnostics Sector Imager 6000.

### Clinical trial data

Samples from the LILACS (Low-dose interleukin-2 in patients with stable ischemic heart disease and acute coronary syndrome; NCT03113773) clinical trial (22) permitted ILC2 fluorescence-activated cell sorting analysis on banked peripheral blood mononuclear cells (PBMCs) using a LSRII Fortessa cytometer. The trial received a favorable evaluation from the Greater Manchester Central Research Ethics Committee, UK (17/NW/0012). Circulating eosinophil count was performed using a Sysmex automated analyzer.

### Statistical analysis

Statistical analysis was performed using GraphPad Prism 7 (Graph Pad Software). Mann-Whitney *U* test was used for nonparametric datasets or 2-way analysis of variance where appropriate. *P* values are indicated in individual figure legends. Error bars represent ± SEM.

## Results

### ILC2s expand in pericardial adipose tissue after MI in an ST2-dependent manner

On D0, D1, D3, D5, and D7 post-MI, mice were killed and the proportions of ILC2 were measured in heart, pericardial adipose tissue (PcAT), and perigonadal adipose tissue (GWAT) by flow cytometry (Supplemental Figure 1A). ILC2 populations in heart and PcAT (Supplemental Figure 2A) were proportionally higher than normally found in peripheral lymph nodes or spleen and observations of ST2 expression suggests cardiac-infiltrating ILC2s are similar to tissue resident ILC2s found in GWAT, whereas the lower ST2 expression in PcAT more closely resembles those found in the periphery (Supplemental Figure 2B).

**Figure 1:**
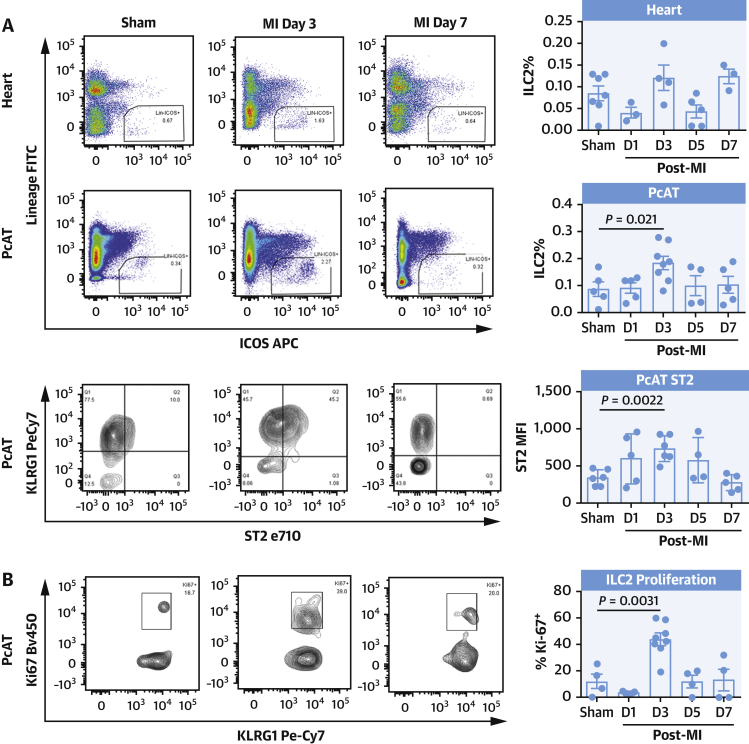

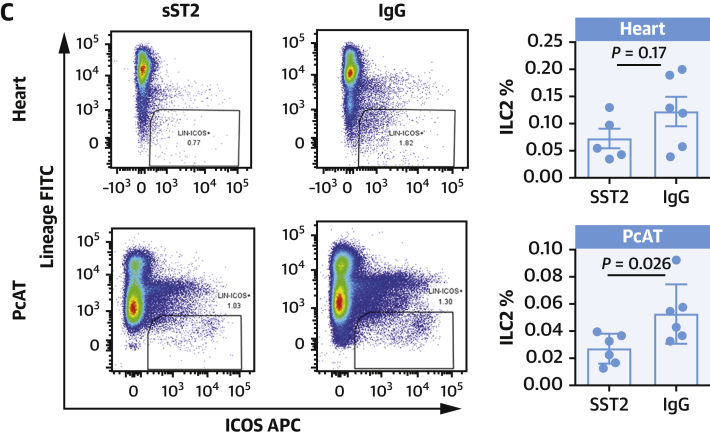
ILC2s Proliferate in PcAT After MI Flow cytometric analysis detects the expansion of ILC2 3 days post-MI **(A)** in heart **(top)** and pericardial adipose tissue accompanied with elevated ST2 surface expression **(bottom)**, and increased proliferation (Ki67) **(B)**. ILC2 proliferation in the PcAT was inhibited by injections of sST2, less pronounced in the heart **(C)**. Representative images shown. Mann-Whitney *U* test. ILC2 = type 2 innate lymphoid cells; MI = myocardial infarction; PcAT = pericardial adipose tissue; sST2 = soluble ST2.

In the PcAT, the ILC2 population expanded 2-fold on D3 before returning to presurgery levels (Figure 1A) while heart resident ILC2s were largely unchanged. During this period, ST2 expression within the PcAT compartment increased stepwise with the expansion of ILC2s, before regressing to its initial expression level by D7 (Figure 1A). Intracellular staining for proliferation marker Ki67 in PcAT-resident ILC2s showed that there was a 3-fold increase in the proliferating population at the D3 time point (Figure 1B), suggesting that the increase in cell number at this time point was due in part to local proliferation.

In the context of MI, it is possible that release of IL-33 from damaged myocardium was driving the proliferation of ST2^+^ ILC2s in the inflamed tissues. To address this, mice were treated with 10 mg/kg sST2 or isotype control the day before MI surgery (D0), as well as D1 to D3, and killed on D3. Flow cytometric analysis showed that in PcAT, there was a significant (*P* = 0.026) decrease in the abundance of ILC2 present in the group receiving sST2 compared with immunoglobulin G control, with a similar trend in heart-infiltrating cells, indicating that IL-33 likely drives the expansion of ILC2s after MI. These data taken together provide evidence that ILC2s are part of the orchestrated response to cardiac tissue damage.

### Genetic ablation of ILC2 impedes the recovery of heart function after MI

**Figure 2:**
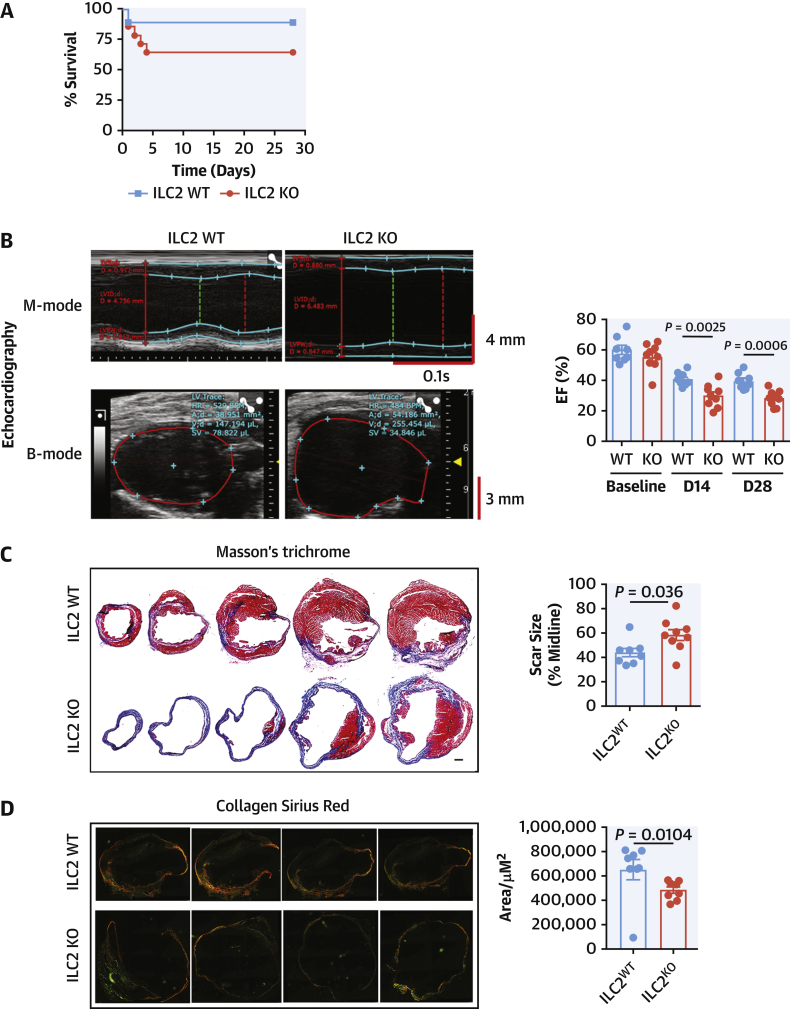
Diminished Cardiac Function in ILC2^KO^ Mice After MI **(A)** Survival curve for knockout (KO: *Rora*^*fl/fl*^*Cd127*^*Cre/+*^, n = 5/14 ruptured) compared with wild type (WT: *Rora*^*fl/fl*^*Cd127*^*wt*^, n = 1/9 ruptured) mice after experimentally induced MI (*P* = 0.2). **(B)** A significant impact on cardiac function (% ejection fraction, EF) in KO mice at Day 14 post-MI (*P* = 0.0025) (representative images shown) and recovery was severely impacted up to 4 weeks post-surgery (*P* = 0.0006). **(C)** WT mice maintained more cardiac muscle compared with KO and had significantly smaller scars. **(D)** Further, Sirius red labeling demonstrates decreased collagen deposition in the scar tissue of KO mice. Mann-Whitney *U* test. Abbreviations as in Figure 1.

Previously, we have shown that genetic ablation of ILC2 in *Rora*^*fl/fl*^ *Cd127*^*cre*^ mice was sufficient to increase atherosclerotic burden during high-fat feeding (14). Using these mice in the LAD ligation MI model, we observed a trend toward a difference in mortality between ILC2 replete (ILC2^WT^) and ILC2 knockout (ILC2^KO^) cohorts (Figure 2A). The surviving mice were followed for 4 weeks with echocardiography (Figure 2B), and during this period, ILC2^KO^ mice had consistently poorer heart function compared with ILC2^WT^ mice. By D14 post-MI, cardiac function (% ejection fraction) had fallen by a mean of 41.04% vs a decrease of 30.03% in ILC2^KO^ and ILC2^WT^ mice, respectively.

Four weeks after MI, scar size was significantly larger in in ILC2^KO^ mice compared with controls (Figure 2C). Sirius red staining of the sections revealed that although the scars were larger, the per unit area of collagen in each section (Figure 2D) was significantly lower in ILC2^KO^ mice. Together, these findings suggest that ILC2s play a positive role in the early response to MI, slewing scar formation toward less intrusive remodeling in the proceeding recovery period.

**Figure 3:**
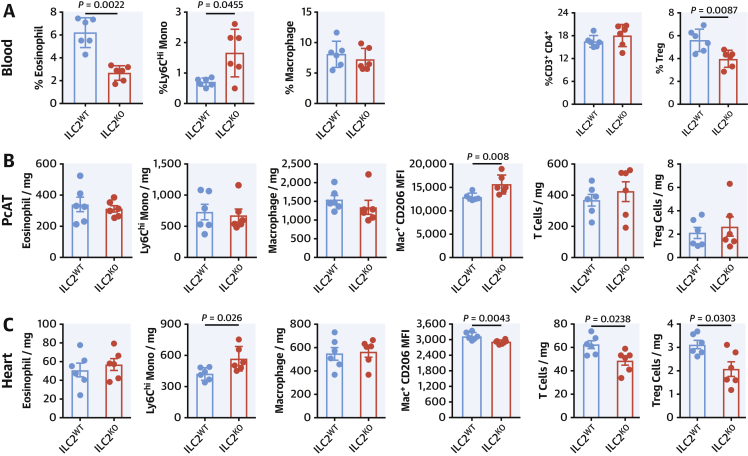
Alterations to the Myeloid Compartment Following MI Cells from heart **(A)**, pericardial adipose tissue (PcAT) **(B)**, and blood **(C)**. Eosinophils, monocytes, macrophage, and Treg populations were characterized by flow cytometry at Day 5 post-MI. Mann-Whitney *U* test. Treg = T regulatory cell; other abbreviation as in Figure 1.

We performed detailed phenotypic analysis on the infiltrating cells by flow cytometry at D5 post-MI. In the absence of ILC2, circulating eosinophils and Tregs were suppressed while inflammatory Ly6C^Hi^ monocytes were more abundant (Figure 3A). Within the PcAT (Figure 3B), the populations are largely unchanged, although in this tissue the macrophages express more surface CD206. Analysis of the heart-infiltrating myeloid cells (Figure 3C) showed there were no differences in eosinophil or macrophage representation in ILC2-deficient mice; however, the number of infiltrating Ly6C^hi^ inflammatory monocytes were increased and cardiac macrophages expressed less CD206, reflective of a more inflammatory phenotype. Heart-infiltrating T cells and Tregs were fewer in number, although the proportion of Tregs within the population was constant (Supplemental Figure 3).

### IL-2 axis as a major upstream regulator of ILC2s in post-MI

**Figure 4:**
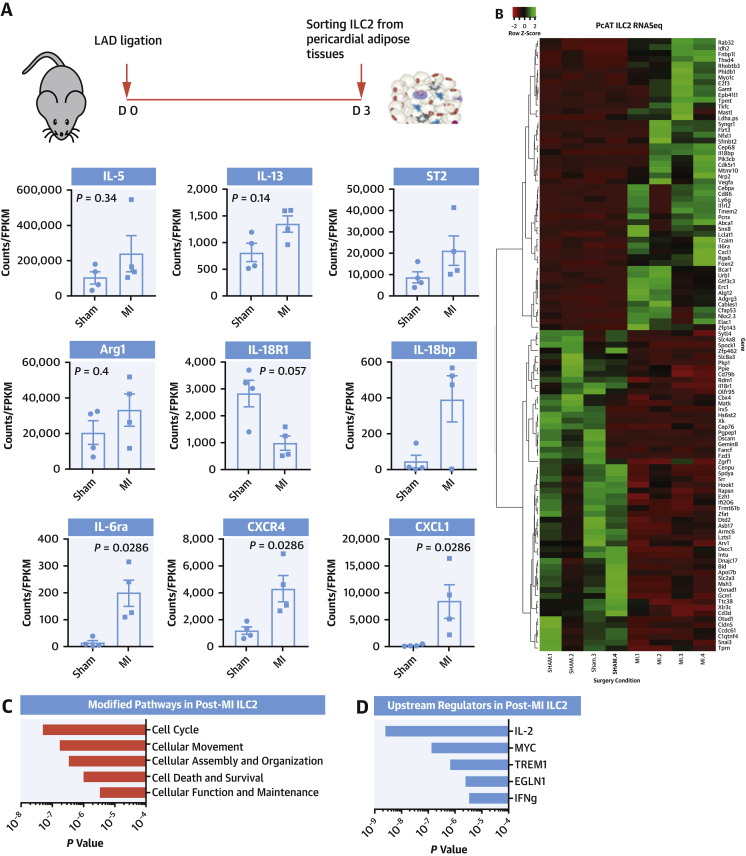
ILC2 Transcriptome After MI Highlight a Potentially Protective IL-2 Stimulus RNAseq analysis of ILC2s sorted from the PcAT on Day 3 post-MI. **(A)** Initial indications of alterations in signature ILC2 transcripts in MI mice as well as significant differences in expression of inflammatory and trafficking molecules. **(B)** Heatmap of in-depth pathway and predictive gene cluster analysis highlights changes in expression of novel genes induced by infarction, which may be central around modulation of cell proliferation and survival pathways **(C)**. **(D)** Predictive upstream regulatory pathways identified an IL-2-driven survival fingerprint. IL = interleukin; RNAseq = RNA sequencing; other abbreviations as in Figure 1.

ILC2s are more abundant in pericardial adipose tissue, which may in this instance be functioning as a reservoir for responding cells that then traffic to the heart. The conditioning these cells receive during the period of inflammation may shed light on cardioprotective regulatory pathways promoted by ILC2. We performed RNAseq analysis of PcAT ILC2 cells sorted from D3 post-MI or from sham mice. Within the sequence data, transcripts typically high in ILC2 (eg, *Rorα, Gata3*, Supplemental Figure 4A) were well represented, whereas those that would be highly expressed in potential “contaminating” cell types (eg, *Cd19, Cd11b*, Supplemental Figure 4B) were relatively rare, confirming sort purity. Approximately 800 differentially expressed transcripts were detected with an adjusted *P* value of <0.05, of those 309 were of known, characterized genes (Supplemental Figure 5). Prominent ILC2 genes (*Il5*, *Il13*, *St2,* and *Arg1*) were maintained or even increased after the onset of inflammation (Figure 4A), and several genes of interest were also identified (Figure 4B). Changes to the IL-18 pathway through decreased expression of *Il18r1* and enhanced *Il18bp* (IL-18 decoy protein) might suggest reinforcement of the protective type 2 pathway. Other genes involved in lymphocyte trafficking signals during MI and remodeling, *Cxcr4* and *Cxcl1*, were increased. Subsequent in silico pathway analysis identified the most likely pathways modified in ILC2 revolve around cell cycle, proliferation, and survival (Figure 4C). Most useful from this analysis was identifying genes linked to certain activation pathways, so-called upstream regulators (Figure 4D). IL-2 was the most well represented pathway (*P* = 2.44 × 10^−9^), key to ILC2 survival, and its receptor CD25 is a defining molecule for the ILC2 population. Others were the MYC pathway (*P* = 1.28 × 10^−7^) is a very well characterized cell survival/growth regulator, TREM1 (*P* = 6.51 × 10^−7^) is an activatory receptor usually found on the surface of myeloid cells that can drive activation through ERK phosphorylation and Ca^++^ mobilization (23); and EGLN1 (*P*= 2.42 × 10^−6^) is a prolyl hydroxylase that suppresses the HIF1α response through both proteolytic degradation and suppression of transcriptional activity (24,25).

### IL-2 axis is central to ILC2 function and MI outcome in mice

**Figure 5:**
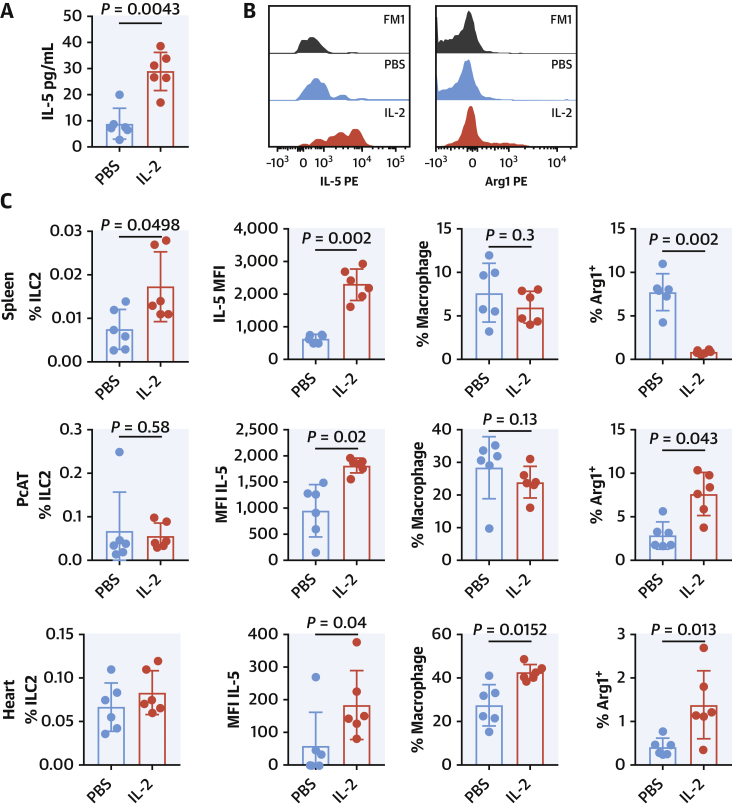
IL-2 Improves ILC2 Function During Acute MI in *Rag2*^*−/−*^ Mice IL-2/Jes6-1 treatment enhances serum cytokine secretion of IL-5 at Day 3 post-MI **(A)**. Intracellular detection of IL-5 (**B, black** histogram FM1, **blue** PBS, and **red** IL-2 treated) complements this. Splenic ILC2s **(C)** were expanded and expressed more IL-5 per cell. Splenic macrophages slew toward Arg1+ population (M2-like). PcAT ILC2s **(C)** were not expanded but expressed more IL-5 with biased macrophage population toward Arg1+ expression. Heart resident ILC2s **(C)** were not expanded but expressed more IL-5 per cell. Subsequent heart-infiltrating macrophages slew toward the Arg1+ population (M2-like). Mann-Whitney *U* test. Arg1+ = Arginase1+; PBS = phosphate-buffered saline; other abbreviations as in Figures 1 and 4.

IL-2 was chosen for its high score, high relevance to ILC2 function, and its potential as a therapeutic modulator of immune responses in patients with acute coronary syndrome (ACS) (22,26). We therefore subjected T-cell–deficient *Rag2*^*−/−*^ mice to LAD ligation and treated them for 3 days with the IL-2/Jes6-1 complex (27) or PBS vehicle (Figure 5). Serum cytokine secretion was enhanced following IL-2/Jes6-1 injection and elevated IL-5 was observed (Figure 5A), although at this time point no change in IL-13 was detected (Supplemental Figure 6A). Complementing this, there was an expansion of peripheral ILC2s in the spleen (Figure 5C), and IL-5 expression in ILC2 was enhanced (Figures 5B and 5C), a pattern also observed in PcAT and in the heart (Figure 5C). Proportions of splenic macrophages were unchanged; however, the expression of the Type II marker Arginase 1 (Arg1) was decreased. Conversely, within the PcAT macrophage, Arg1 expression was elevated (coinciding with more CD206 in this case, Supplemental Figure 6B) slewing the population toward a wound-healing phenotype. Macrophages were also detected within the heart tissue in greater abundance, and this was accompanied by a greater proportion of them expressing Arg1 (Figures 5B and 5C).

**Figure 6:**
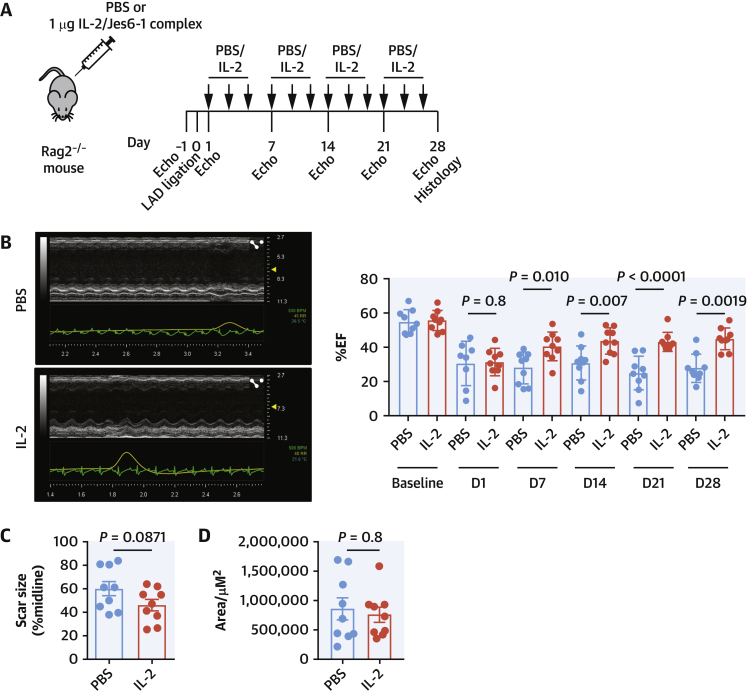
IL-2 Treatment Improves the Recovery of Heart Function After MI **(A)***Rag2*^*−/−*^ mice (n = 9 per group) were subjected to LAD ligation and treated intraperitoneally 3 times a week with the IL-2/Jes6-1 complex or PBS vehicle alone (see Methods). IL-2 treatment substantially improves cardiac recovery after MI as shown by echocardiography **(B)** and the significant difference in % ejection fraction (%EF) between the 2 groups of mice by the primary endpoint at Day 28, despite no difference in infarct size **(C)** or collagen deposition (**D**). Mann-Whitney *U* test or 2-way analysis of variance where appropriate. Abbreviations as in Figures 1 and 4.

Assessing the impact of these phenotypic changes on long-term MI outcome, *Rag2*^*−/−*^ mice received 3 weekly injections of IL-2/Jes6-1 or PBS vehicle for 4 weeks (Figure 6A) accompanied by regular assessment of cardiac function. There were no immediate differences between the IL-2 and PBS cohorts; however, distinct improvement in cardiac function was observed in the IL-2-treated cohort after 7 days (Figure 6B). This improvement was maintained for the duration of the experiment, increasing to 45% by week 4 (*P* = 0.0012) compared with 28% in the PBS cohort (Figure 6B), despite no difference in infarct size (Figure 6C) or collagen deposition (Figure 6D). No alterations to macrophage infiltrate or apoptosis (via TUNEL staining) were observed at this time (data not shown).

### Low-dose IL-2 influences ILC2 behavior in patients with ACS

**Figure 7:**
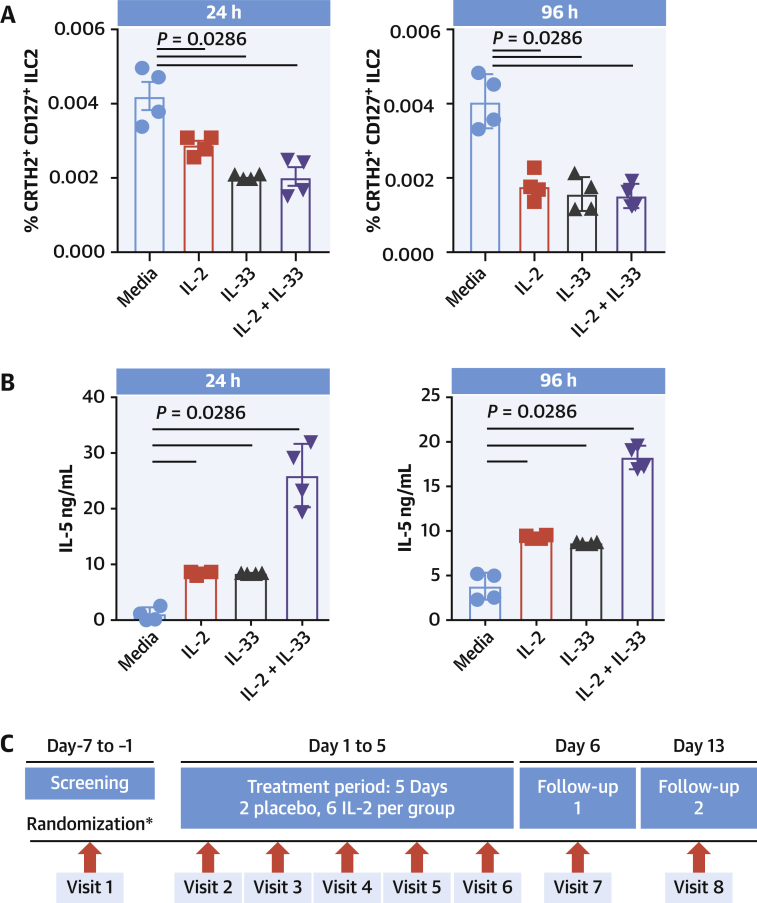

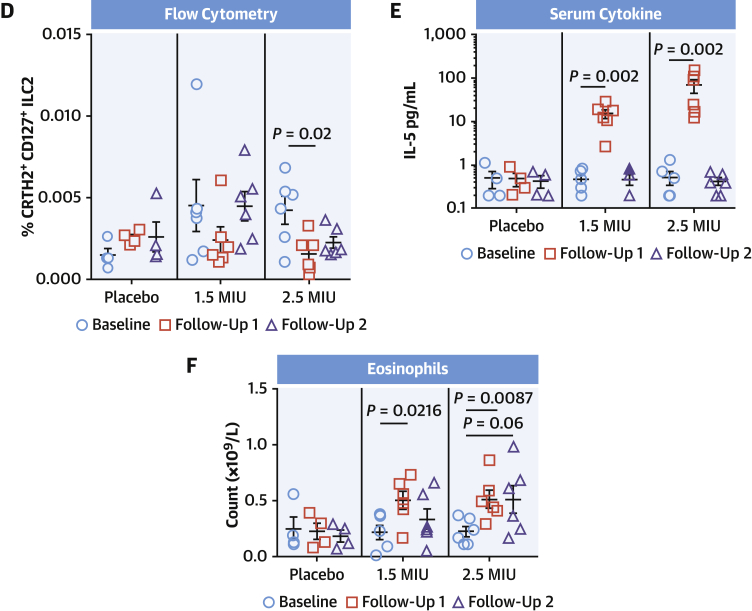
Low-Dose IL-2 Activates ILC2s in Patients With ACS PBMC culture with IL-2 (10 ng/mL) show rapid downregulation of ILC2 surface markers following IL-2, IL-33, and in combination **(A)**. Secretion of IL-5 was increased **(B)**. Patients with ACS enrolled in LILACS **(C)** were sampled at Visit 2 (Baseline) before receiving 5 daily consecutive IL-2 doses (1.5 U or 2.5 U) or placebo and follow-up before blood sampling on Visit 7 (Follow-up 1, Day 6) and Visit 8 (Follow-up 2, Day 13). **(D)** Flow cytometric analysis shows a dose-dependent decrease in canonical markers of peripheral blood ILC2 (CRTH2^+^CD161^+^ cells within Lin^−^CD127^+^ cells) over the course of the treatment, suggesting activation. Corresponding amounts of serum IL-5 **(E)** and blood eosinophil count **(F)** were both elevated at follow-up 1 and subsequently decreased after a further 6 days. **Error bars** show ± SEM. Multiple comparisons were made using the Kruskal-Wallis test followed by Mann-Whitney *U* test. ACS = acute coronary syndrome; PBMC = peripheral blood mononuclear cell; other abbreviations as in Figures 1 and 4.

So far, we have shown that in mouse models of MI, an ILC2/IL-2 axis is beneficial to improved recovery of cardiac function. Repurposing recombinant IL-2 (aldesleukin) for treating human cardiovascular disease is of interest, although currently its licensed indication is limited to metastatic melanoma and metastatic renal cell carcinoma. In humans, IL-2 has been shown to activate ILC2 in vitro, which is associated with reduced expression of canonical markers CRTH2 and CD127 (28), potentially masking the presence and importance of this population. To address this, we studied the effect of IL-2 treatment on whole PBMCs and how it modified the ILC2 population. PBMCs isolated from donor blood was cultured with 10 ng/mL IL-2, IL-33, or in combination for 24 to 96 hours, and any changes to ILC2s were monitored by flow cytometry. Here, ILC2s rapidly lost their surface phenotype during treatment (Figure 7A) in association with increased capacity to secrete IL-5 (Figure 7B), indicating an activation status.

The LILACS trial was designed to evaluate the safety and pharmacodynamics of low-dose IL-2 in patients with ACS. After recruitment, individuals received repeated blinded daily doses of IL-2 (1.5 × 10^6^ IU, n = 6 patients or 2.5 × 10^6^ IU, n = 6 patients) or placebo (n = 4 patients) over a period of 5 days, with follow-up sampling on D6 and D13 (Figure 7C). Flow cytometry analysis of PBMCs from this trial showed that although placebo had no effect on ILC2 populations, patients receiving 1.5 MIU/d of IL-2 for 5 consecutive days showed a trend toward a decrease in ILC2 canonical markers CRTH2 and CD127 immediately after the treatment course (follow-up 1), which then recovered after 1 week (follow-up 2) (Figure 7D). Patients receiving the higher 2.5 MIU/d dose for 5 consecutive days showed a significant decrease in ILC2 canonical markers immediately after the treatment course, which remained low 1 week later (Figure 7D). These features are indicative of ILC2 activation on treatment with low-dose IL-2 (28). Serum IL-5 titers and peripheral eosinophil counts were elevated in both treatment groups after low-dose IL-2 in a dose-dependent manner and both subsequently returned to basal levels after 1 week (Figures 7E and 7F), further supporting an IL-2-driven activation of circulating ILC2s.

## Discussion

Here, we have shown that ILC2s have a direct and protective role in recovery from experimental MI. ILC2s were detected in the heart and pericardial adipose tissue in steady state and expanded during the initial inflammatory phase of MI, trafficking from the blood under some conditions. The expansion within the inflamed tissues is rapid but not sustained and falls away within the first week of recovery. This complements earlier observations that there is a cardiac resident population of ILC2s that are present in the heart during periods of pericarditis and MI (16), and we show that cardiac remodeling and wound stability is severely compromised in their absence. As we have seen in other models of ILC2 response to tissue damage (eg, atherosclerosis), the types of wounds that develop in the absence of ILC2s tend to be larger, have less collagen deposition, and are overall more fragile than ILC2-replete mice (14); as a consequence, the trend toward higher mortality rate seen in ILC2^KO^ mice during experimental MI was due to cardiac rupture.

Transcript analysis highlighted 2 main signatures of ILC2 activation during MI in the PcAT: proliferation and trafficking. The expression of CXCR4 would allow the ILC2 to be recruited to the inflamed tissue in the heart through interaction with CXCL12 (29) (known to increase during ischemia [30]) or MIF (31). The suppression of the IL-18 axis through decreased expression of IL18R and increased IL18BP (which sequesters IL-18 [32,33]) may reinforce the polarization of ILC2 and potentially nearby T cells, reducing the opportunity for plasticity. This is consistent with the trend toward reduced accumulation of Tregs in the hearts of ILC2-deficient mice, but more work is needed to fully describe the mechanisms.

Pathway analysis of the RNAseq dataset provided a useful insight of what may be triggering the ILC2 response, and identification of IL-2 and other novel upstream regulatory pathways could also provide useful mechanisms to modify ILC2 behavior during the acute phase of MI.

The results observed during the direct targeting of ILC2 with IL-2/Jes6-1 complexes were promising, not only preventing a marked decrease in cardiac function during the initial phase but also improving recovery over the subsequent 4 weeks.

Data from our LILACS human clinical trial provide translational relevance of our preclinical findings. We demonstrate that low-dose IL-2 activates ILC2s in patients presenting with ACS, with increases in IL-5 and eosinophil levels as a consequence of ILC2 activation (34). Interestingly, recent data in mice have shown that eosinophils may play a significant role in MI, and their deficiency promoted adverse cardiac remodeling (35).

### Study limitations

#### Basic and preclinical results

Our data suggest that the protective effects of ILC2 after MI could be mediated through the production of the type 2 cytokine IL-5; however, the direct role of ILC2-derived IL-5 (and downstream eosinophil activation) in mediating this protective effect will require further investigations. We used a permanent coronary artery ligation model to create large infarcts. The role of ILC2 in the response to ischemia-reperfusion injury will require further studies.

#### Human clinical results

Our data show increased serum IL-5 and blood eosinophil counts after low-dose IL-2, in association with signs of ILC2 activation; however, the potential protective role of such changes will require further investigation.

## Conclusions

We have presented data that highlight a critical role for ILC2 during the response to MI. Expanding early to condition the immune response, their absence increases the accumulation of inflammatory monocytes and macrophages and severely impacts cardiac function after MI. We have shown that expansion of ILC2 with exogenous IL-2 is beneficial, driving heart macrophages toward an alternatively activated and potentially reparative phenotype and improving cardiac output for the duration of the trial (Central Illustration). Future work should address the precise mechanisms through which ILC2s control the inflammatory and cardiac remodeling response after MI. Early translational data of low-dose IL-2 in patients with ACS suggest that this may promote ILC2 activation, potentially impacting on long-term cardiac dysfunction. Furthermore, acute MI may drive the progression of atherosclerosis (36), which clinically results in increased rate of further atherosclerotic cardiovascular events in the intervening period. Therefore, one can hypothesize that targeting ILC2s may be beneficial not only in cardiac remodeling post-MI but potentially also retards progression of atherosclerosis.

Perspectives **COMPETENCY IN MEDICAL KNOWLEDGE:** Inflammation impedes cardiac remodeling and recovery of ventricular function after MI. IL-2 plays a critical role in ILC2 activation and myocardial repair.

**TRANSLATIONAL OUTLOOK:** Clinical trials of low-dose IL-2 are needed to evaluate therapeutic efficacy in patients with acute MI.

**Central Illustration:**
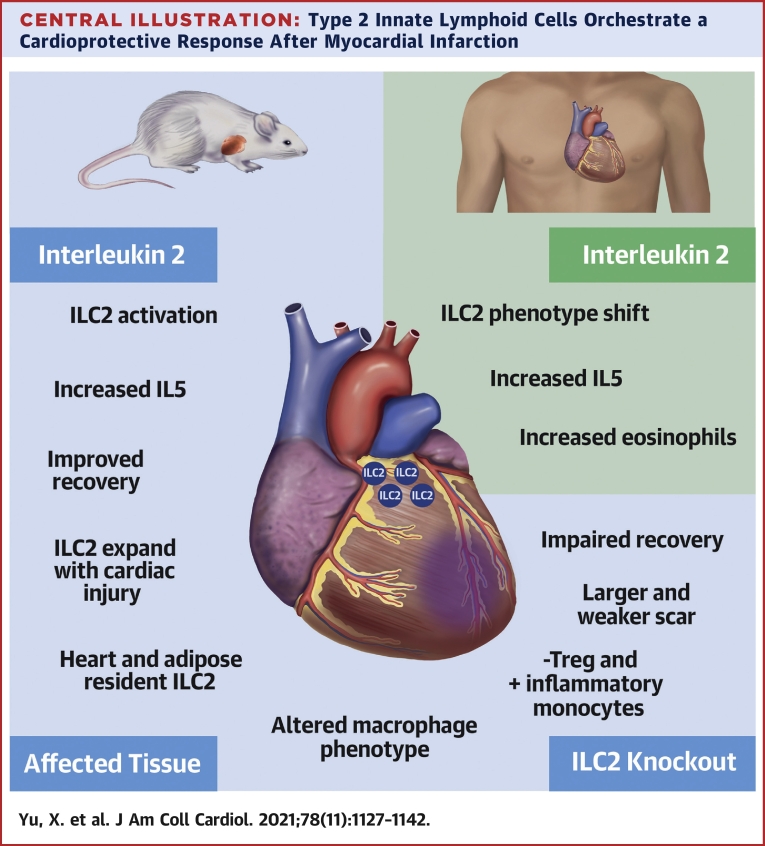
Type 2 Innate Lymphoid Cells Orchestrate a Cardioprotective Response After Myocardial Infarction In this report, we demonstrate that type 2 innate lymphoid cells (ILC2s) are resident in the heart and pericardium, expanding during experimental myocardial infarction. In mouse models deficient in ILC2s, cardiac recovery and repair are significantly retarded; the recovering scar contains less collagen, fewer T regulatory cells (Tregs) and more inflammatory monocyte/macrophage populations. Supplementing the mice with interleukin (IL)-2 increases ILC2 activation and promotes the secretion of cardioprotective cytokines (eg, IL-5) and ultimately improves recovery. We observe a similar phenotypic shift in ILC2s in patients with acute coronary syndromes after administration of low-dose IL-2, coupled with increased IL-5 secretion and associated eosinophilia.

## Funding Support and Author Disclosures

This research was supported by the Cambridge NIHR BRC Cell Phenotyping Hub. Dr Newland has received funding from the British Heart Foundation (PG/18/20/33595). Dr Yu has been funded by a Royal Society Newton Advanced fellowship (NA140277). The work received support from European Research Area Network on Cardiovascular Diseases Joint Transnational Call (ERA-CVD JTC) 2017-PLAQUEFIGHT). Dr Sun has been funded by the Federation Française de Cardiologie. The LILACS trial was funded by the Medical Research Council, grant number MR/N028015/1, the British Heart Foundation Cambridge Centre of Excellence (RCAG/521). Mr Cheriyan has received funding support from the NIHR Cambridge Biomedical Research Centre (BRC-1215-20014). The views expressed are those of the authors and not necessarily those of the NIHR or the Department of Health and Social Care. Dr Rudd has been part-supported by the NIHR Cambridge Biomedical Research Centre, the British Heart Foundation, HEFCE, the EPSRC, and the Wellcome Trust. This NGS work was performed with the Genomics and Transcriptomics Core, which is funded by the UK Medical Research Council (MRC) Metabolic Disease Unit (MRC_MC_UU_00014/5) and a Wellcome Trust Major Award (208363/Z/17/Z). All authors have reported that they have no relationships relevant to the contents of this paper to disclose.
