# Supplementary material for: Innate Lymphoid Cells Promote Recovery of Ventricular Function After Myocardial Infarction
Source: J Am Coll Cardiol. 2021 Sep 14;78(11):1127–42. doi: 10.1016/j.jacc.2021.07.018 (PMC8434674; doi:10.1016/j.jacc.2021.07.018)
Supplement: Supplemental Data [file mmc1.docx]

**Online Appendix**
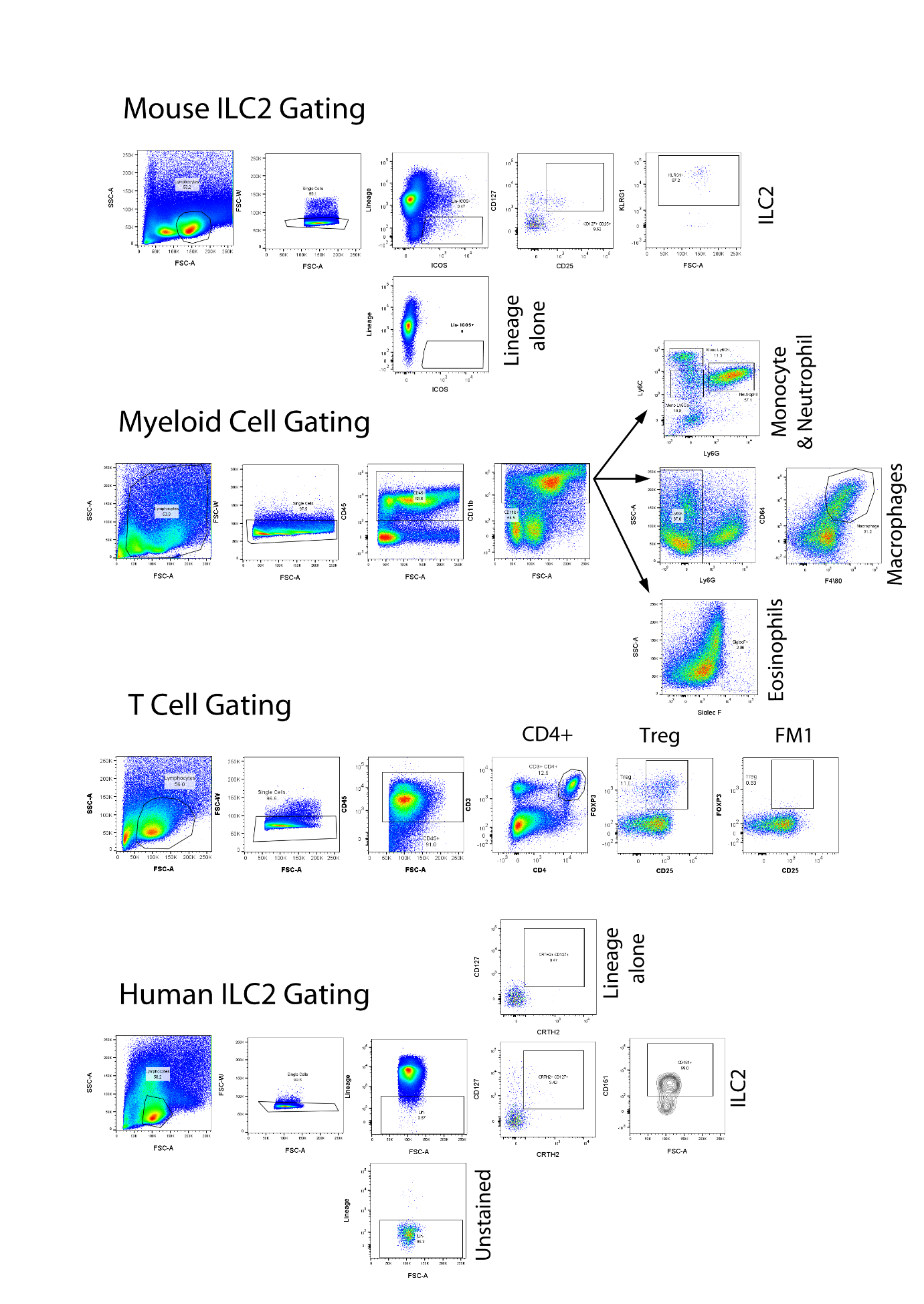


**Supplemental Figure 1: Examples of hierarchical Flow cytometry gating**

Example flow cytometry gating identifying Immune cell populations described throughout this manuscript. Top, Mouse ILC2 cells, defined as Live single cells Lineage^-^ ICOS^+^ CD25^+^ CD127^+^ KLRG1^+^ ST2 ^variable^. Secondary gating set using lineage alone.

Middle top, Myeloid populations were co-stained and defined universally as live single cells CD45^+^ CD11b^+^. Subsequent populations of Monocytes (Ly6G^-^, Ly6C^variable^), Neutrophils (Ly6C^lo^, Ly6G^+^), Macrophages (Ly6G^-^ F4\80^+^ CD64^+^) and Eosinophils (SiglecF^+^) were gated as shown.

Middle bottom: Regulatory T cell populations were defined as Live single cells CD45^+^ CD3^+^ CD4^+^ CD25^+^ Foxp3^+^ where the expression of Foxp3 was determined by gating on Foxp3 negative in a pooled sample FM1.

Bottom, Human peripheral blood ILC2. Similar to mouse ILC2, human ILC2 are Lineage negative and are identified as follows, Lineage^-^ CD127^+^ CRTH2^+^ CD161^+^. Gating of CD127^+^ CDTH2^+^ were set using a Linage-alone FM1.


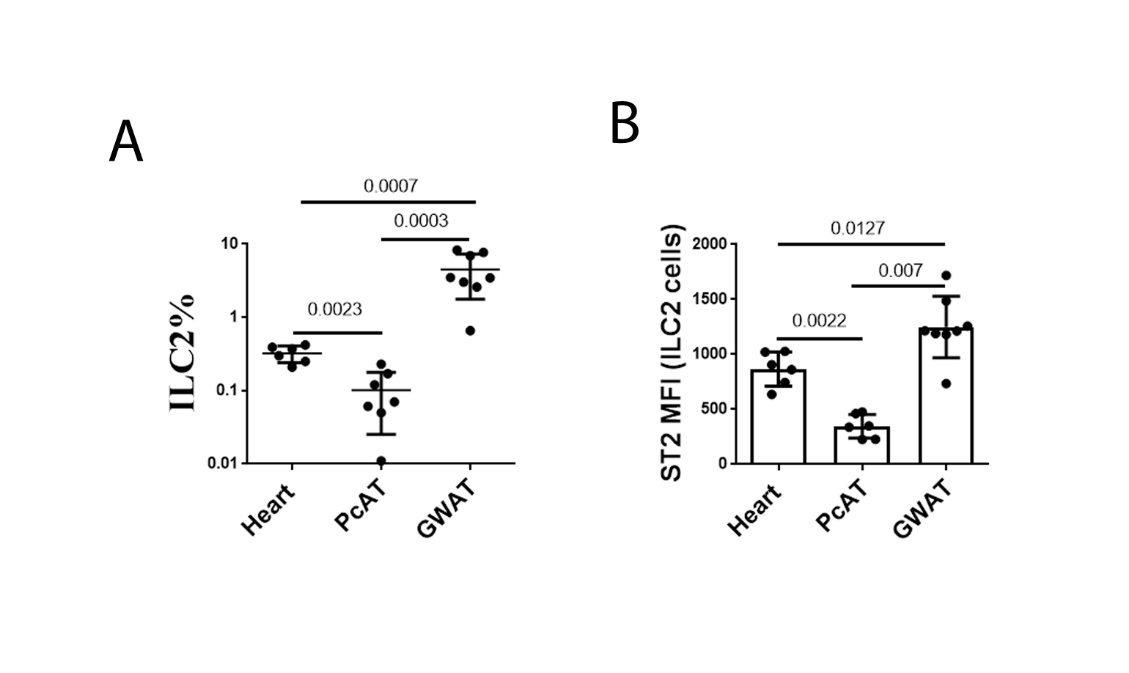


**Supplemental Figure 2. Populations of tissue resident ILC2 in mouse model of myocardial infarction.** ILC2 are present in heart and pericardial adipose tissue (PcAT) (**A**) between 0.1 and 0.5% of CD45^+^ cells respectively, considerably higher than normally found in peripheral lymph nodes (approx. 0.01%) and more similar to GWAT. Heart infiltrating ILC2 express more IL33 receptor ST2 on their surface than PcAT resident again mirroring GWAT resident ILC2 (**B**)


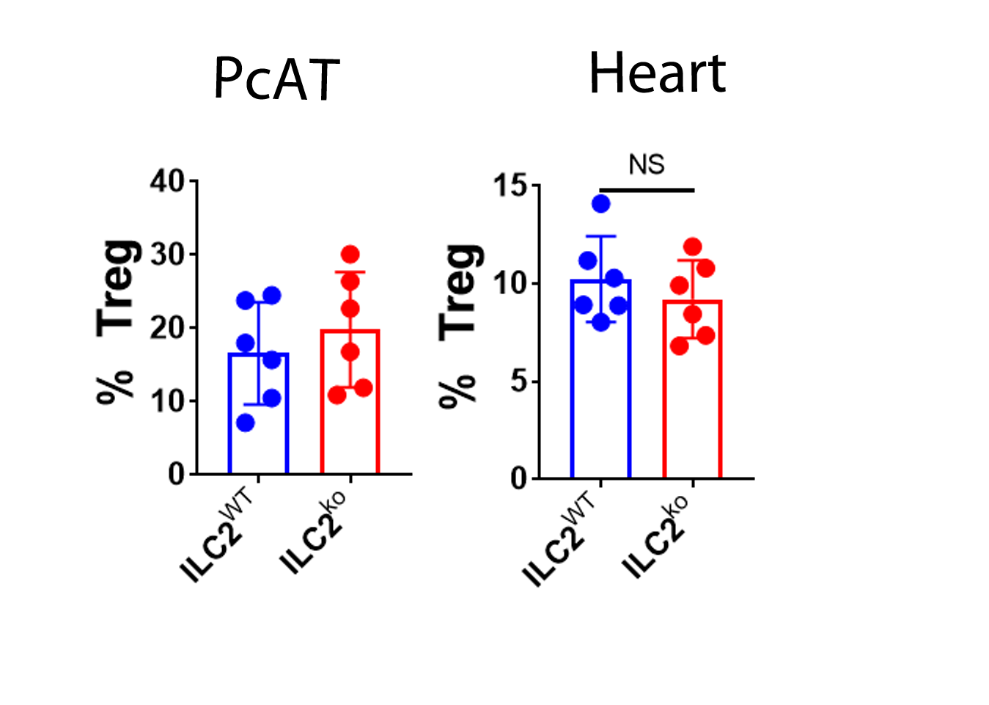


**Supplemental Figure 3: Myocardial infarction does not alter the proportion of Treg cells**. Although there is a numerical decrease in heart infiltrating Treg during LAD induced myocardial infarction, the proportions of T reg cells as % of CD3^+^ CD4^+^ T cells does not alter in ILC2^ko^


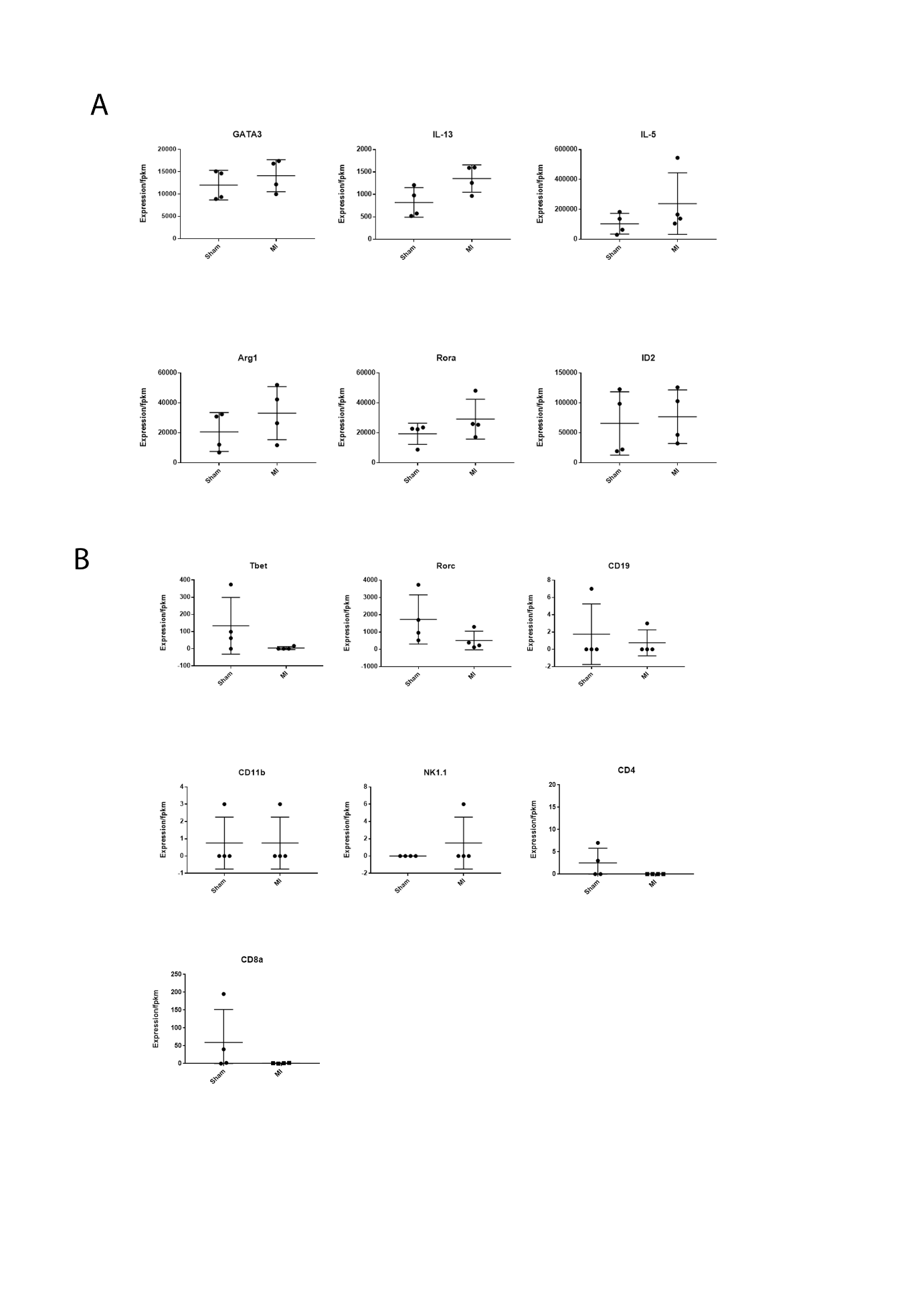


**Supplemental Figure 4: RNAseq analysis of PcAT resident ILC2 3 days after MI surgery.** ILC2 sorted from PcAT on D3 post MI and subject to RNAseq. Relative expression (FPKM) of “typical” ILC2 transcripts *Gata3, Il13, Il5, Arg1, Rora* and *ID2* were well represented in the samples (**A**) whereas transcripts highly expressed in non-ILC2 cell types (*Tbx21, Rorc, CD19, CD11b, NK1.1, CD4* and *CD8)* (**B**) were much less abundant, indicating suitable purity during the sort


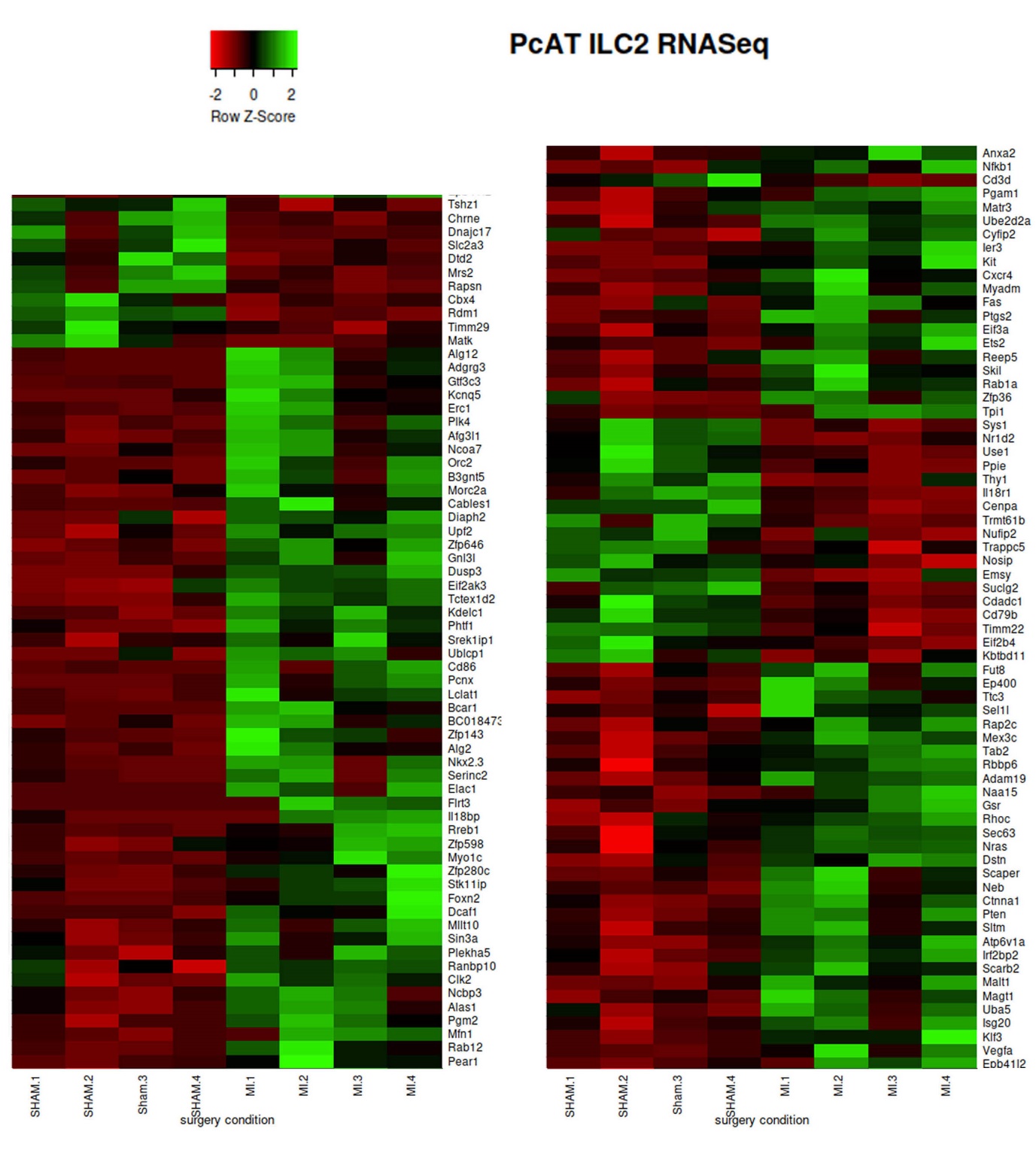


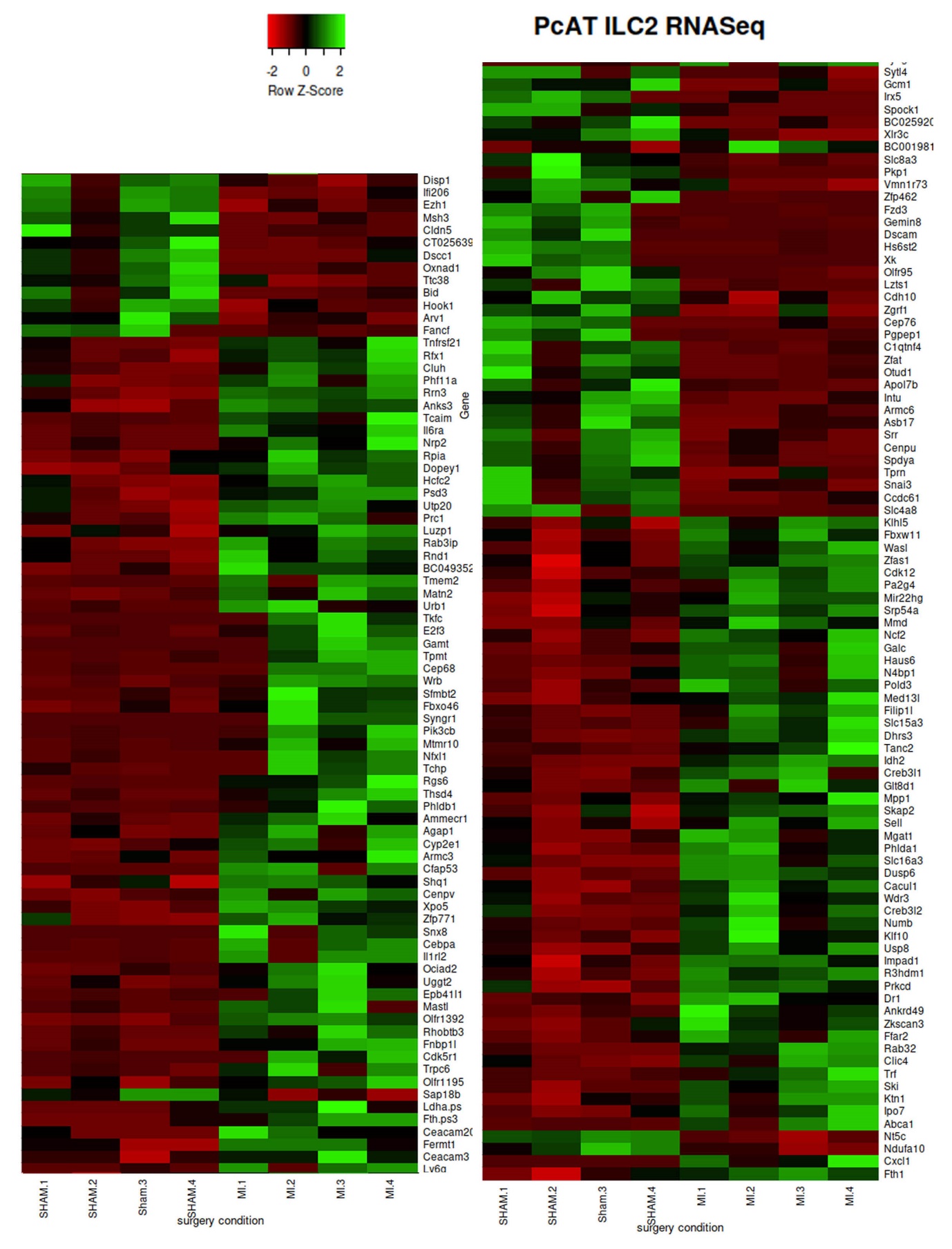


Supplemental Figure 5 continued

**Supplemental Figure 5: Heatmap of RNAseq comparison of sham surgery vs LAD MI.** RNAseq analysis of MI modified transcripts in PcAT ILC2 produced a differentially expressed gene list of 309 known/characterised transcripts, shown here as a heat map of upregulated (Green) or down regulated (Red) genes. Clustered by average linkage with Euclidian distance measurement.


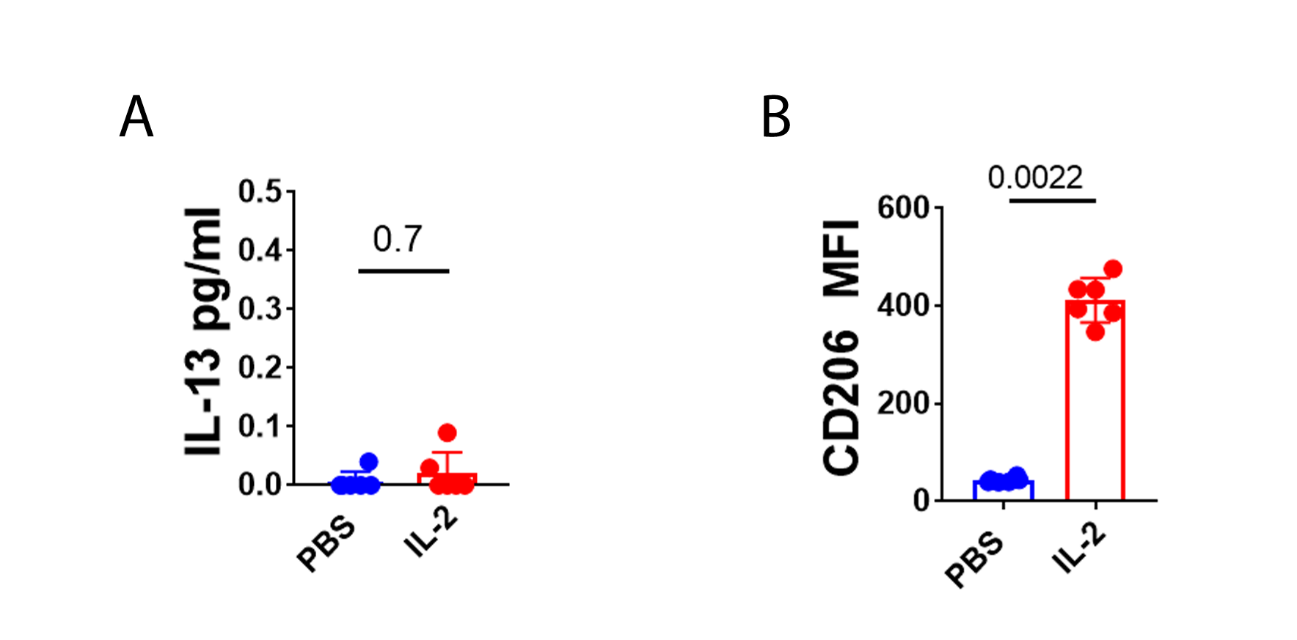


**Supplemental Figure 6: The effect of exogenous IL2 on MI progression.**

Country to the observed increase in IL-5, IL-13 detection remained unchanged in the serum of IL2 treated *Rag2^-/-^* mice (**A**). Further, modification of the macrophage phenotype was observed after IL2 treatment and expression of CD206 on the surface of macrophages is enhanced (**B**) indicative of alternative activation.

**Supplemental Table 1: List of flow cytometry antibody cocktails used for immune cell population analysis.**

| Cell Type | Surface Markers | Antibody (clone) |
| --- | --- | --- |
| Mouse Lineage Cocktail | CD3, CD4, CD19, CD11b, CD11c, NK1.1, Ly6G, Fcer1, Ter119 (Erythrocytes) | CD3 (145-2C11) CD4 (RM4-5) CD19 (1D3) CD11b (M1/70) CD11c (N418) NK1.1 (PK136) Ly6G (Gr1) FcER1 (MAR1) Ter119 (Ter119) |
| Mouse ILC2 | Lin- ICOS+ CD127+ CD25+ KLRG1+ ST2 variable | ICOS (C398.4A) CD127 (eBio3C7) CD25 (3C7) KLRG1 (2F1) ST2 (RMST2-2) |
| Mouse ILC2 Cytokine | Lin- ICOS+ CD127+ CD25+ KLRG1+ ST2 variable IL-5 variable IL-13 variable | ICOS (C398.4A) CD127 (eBio3C7) CD25 (3C7) KLRG1 (2F1) ST2 (RMST2-2) IL-5 (TRFK5) IL13 (eBio13A) |
| Mouse T cell | CD45+ CD3+ CD4+ CD25+ Foxp3+ | CD45 (30F11) CD3 (145-2C11) CD4 (RM4-5) CD25 (3C7) |
| Mouse Macrophage | CD45+ CD11b+ Ly6G- CD64+ F4\80+ | CD45 (30F11) CD11b (M1/70) Ly6G (1A8) CD64 (X54-5/7.1) F4\80 (MCA497APCT) |
| Mouse Macrophage Polarisation | CD45+ CD11b+ Ly6G- CD64+ F4\80+ CD206 variable Arg1 variable INOS variable | CD45 (30F11) CD11b (M1/70) Ly6G (1A8) CD64 (X54-5/7.1) F4\80 (MCA497APCT) CD206 (C068C2) Arg1 (IC5868P) iNOS (CXNFT) |
| Mouse Monocyte | CD45+ CD11b+ Ly6G- Ly6C variable | CD45 (30F11) CD11b (M1/70) Ly6G (1A8) Ly6C (AL-21) |
| Mouse Neutropihl | CD45+ CD11b+ Ly6G+ | CD45 (30F11) CD11b (M1/70) Ly6G (1A8) |
| Mosue Eosinophil | CD45+ CD11b+ SiglecF+ | CD45 (30F11) CD11b (M1/70) SiglecF (E50-24.40) |
| Proliferation | Cell population Ki67+ | Ki67 (SolA15) |
| Human Lineage Cocktail | CD1a, CD3, CD4, CD19, CD14, CD16, CD11c, Fcer1, CD303, CD123, CD34, CD94, TCRa/b, TCR g/d | CD1a (HI149) CD3 (UCHT1) CD4RPA-T4 CD19 (HIB19) CD14 (M5E2) CD16 (3G8) CD11c (3.9) Fcer1 (AER-37) CD303 (BDCA-2) CD123 (6H6) CD34 (581) CD94 (DX22) TCR a/b (IP26) TCR g/d (B1) |
| Human ILC2 | Lin- CD45+ CRTH2+ CD127+ CD161+ | CD45 (2D1) CRTH2 (BM16) CD127 (eBioRDR5) CD161 (HP-3G10) |
